# Cross-Attraction between an Exotic and a Native Pine Bark Beetle: A Novel Invasion Mechanism?

**DOI:** 10.1371/journal.pone.0001302

**Published:** 2007-12-12

**Authors:** Min Lu, Daniel R. Miller, Jiang-Hua Sun

**Affiliations:** 1 State Key Laboratory of Integrated Management of Pest Insects and Rodents, Institute of Zoology, Chinese Academy of Sciences, Beijing, China; 2 Southern Research Station, United States Department of Agriculture Forest Service, Athens, Georgia, United States of America; 3 Graduate School, Chinese Academy of Sciences, Beijing, China; University of Zurich, Switzerland

## Abstract

**Background:**

Aside from the ecological impacts, invasive species fascinate ecologists because of the unique opportunities that invasives offer in the study of community ecology. Some hypotheses have been proposed to illustrate the mechanisms that allow exotics to become invasive. However, positive interactions between exotic and native insects are rarely utilized to explain invasiveness of pests.

**Methodology/Principal Findings:**

Here, we present information on a recently formed association between a native and an exotic bark beetle on their shared host, *Pinus tabuliformis*, in China. In field examinations, we found that 35–40% of *P. tabuliformis* attacked by an exotic bark beetle, *Dendroctonus valens,* were also attacked by a native pine bark beetle, *Hylastes parallelus*. In the laboratory, we found that the antennal and walking responses of *H. parallelus* to host- and beetle-produced compounds were similar to those of the exotic *D. valens* in China. In addition, *D. valens* was attracted to volatiles produced by the native *H. parallelus*.

**Conclusions/Significance:**

We report, for the first time, facilitation between an exotic and a native bark beetle seems to involve overlap in the use of host attractants and pheromones, which is cross-attraction. The concept of this interspecific facilitation could be explored as a novel invasive mechanism which helps explain invasiveness of not only exotic bark beetles but also other introduced pests in principle. The results reported here also have particularly important implications for risk assessments and management strategies for invasive species.

## Introduction

Invasive species have long fascinated ecologists and invasive biologists, not only because they can cause tremendous destruction, but also because we do not yet understand fully how they can successfully invade novel communities [1]–[3]. To curb the future economic and environmental impacts of invasive exotic species, we need to understand the invasion mechanisms behind invasive species. Understanding such mechanisms also might be crucial for the successful management of biological invasions.

Attempts to determine the mechanisms of invasiveness by exotic species have focused primarily on ecological (such as empty niche [4] and enemy release [5]) and evolutionary hypotheses (such as evolution of increased competitive ability [6] and founder events) [7]. In contrast, facilitation between exotic and native invertebrates has received much less attention although the relevance of facilitation may be important, especially when several invasion mechanisms work in synergy [7]–[9]. This may be particularly true for bark beetles (Coleoptera: Scolytidae) that breed in the phloem tissue of coniferous trees, often in multiple-species associations [10].

Competition and facilitation are arguably two of the most important forces in the community ecology of bark beetles. Typically, bark beetles tunnel within phloem tissue, laying eggs along the way [10]. The larvae hatch and feed through the phloem tissue, creating fan or star-shaped galleries. Competition has likely separated different species by host species as well as by host requirements within the same species, resulting in unique niches based on such characters as phloem thickness, tree defense chemistry, nutritional quality and water content [11]. Separation of species within a tree can occur through preference for specific part of a tree (cones, twigs and small branches, large branches, upper and lower trunk, root collar and roots) [10].

Facilitation in bark beetles is evident from concurrent within-tree attacks by secondary species (such as species in the genera *Ips* DeGeer, *Hylastes* Erichson, *Pityogenes* Bedel and *Pityophthorus* Eichhoff) following the activities of aggressive tree-killing bark beetles (such as *Dendroctonus* species) [10], [12]. Attacks by tree-killing species are generally restricted to the main trunks of trees thereby leaving other portions of the same trees (such as upper boles, large branches and twigs) available for other species with little risk of beetle mortality from tree defenses [10]; the trees have already been killed by the aggressive species [12]. These types of facilitated associations may be important in understanding the invasiveness of exotic bark beetles in areas with few competing species of bark beetles and on hosts with little evolutionary experience with aggressive bark beetles.

The most destructive terrestrial invertebrate species to pine forests in China is the exotic red turpentine beetle, *Dendroctonus valens* LeConte [13]. *Dendroctonus valens* was likely introduced into China through the importation of unprocessed logs from the Pacific-Northwest region of the United States [14]–[15]. In 1999, the first major epidemic of *D. valens* began in Shanxi province in northern China, spreading to three adjacent provinces and killing more than 6 million *P. tabuliformis* over an area of 0.5 million hectares in a period of three years [13], [16]–[18].

The aggressive ability of *D. valens* to kill healthy pine trees in China is in distinct contrast to its biology in its native range of North America where *D. valens* favors weakened, dying or fire-scorched trees, rarely killing trees outright but at times weakening them enough for successful attacks by other species of bark beetles [10]. During the course of field studies on *D. valens* in China, the authors noted an unusual prevalence of another bark beetle species, *Hylastes parallelus* Chapuis, in pine forests under attack by *D. valens*. Historically, *H. parallelus* is a secondary bark beetle, common in spruce and pine forests of China, Korea, Russia and Japan, and generally breeding in roots and stems of stressed trees [19]–[20]; *H. parallelus* is not found in North America [21]. It is possible that initial attacks by the exotic *D. valens* are followed closely by infestations by the native *H. parallelus*, leading to facilitation between the two species. Stress in the root system of a pine tree, such as one caused by an infestation by a phloem-feeding species (*H. parallelus*), could increase the attack success of a primary species such as *D. valens*. Elevated levels of stress caused by an increase in *D. valens* attack success could also enhance the breeding opportunities for *H. parallelus*. Instances of tree mortality involving *D. valens* in North America have involved attacks by both primary and secondary bark beetles [10].

Cross-attraction of bark beetle species is fairly common in North America and Europe, particularly with respect to compounds produced by host trees, such as ethanol and monoterpenes, and pheromones, such as ipsenol and ipsdienol [22]–[23]. Pre-adaptations in chemical communication may facilitate mutualistic associations with exotic species [24]. Therefore, we evaluated facilitation of an exotic and a native bark beetle based on field study and antennal and behavioral experiments in the laboratory. Our objectives were threefold: (1) To ascertain the relationship between the exotic *D. valens* and the native *H. parallelus* in the roots of *P. tabuliformis*. (2) To determine antennal and behavioral (walking) responses of *H. parallelus* to host- and beetle-produced compounds. These types of responses have already been determined for *D. valens* in China [25]. (3) To determine antennal and walking responses of *D. valens* to volatiles produced by *H. parallelus.* The results provide the first experimental evidence of a positive interaction between an exotic and a native bark beetle.

## Results

### Field Association between *D. valens* and *H. parallelus*

**Figure 1:**
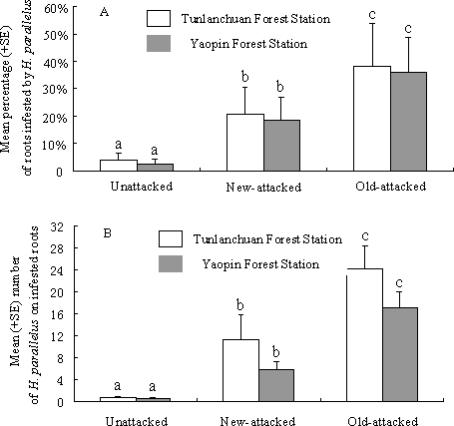
Field association between *D. valens* and *H. parallelus.* Percentage of roots infested by *H. parallelus* (A) and average number of *H. parallelus* on each infested pine root (B) for pine trees unattacked by *D. valens*, newly-attacked by *D. valens*, and old-attacked by *D. valens* at two experimental sites in China (*n* = 10).

We found *H. parallelus* on roots of *P. tabuliformis* at both locations in China, with a significant association with attacks by *D. valens* (ANOVA, *F*_2, 27_ = 68.71, *P*<0.001 at the Tunlanchuan Forest Station; *F*_2, 27_ = 57.41 at Yaopin Forest Station, *P*<0.001). At both locations, healthy pines with no evidence of attacks by *D. valens* had low numbers of roots infested by *H. parallelus* (<0.05%), with approximately one adult beetle on each attacked root (Fig. 1A–B). Pines with recent and old attacks by *D. valens* had significantly more roots with *H. parallelus* than pines unattacked by *D. valens*, with the highest percentage of infested roots on trees with old attacks by *D. valens* (35–40%) (Fig. 1B). The infestation rate of roots in trees with recent attacks by *D. valens* was approximately 20% at both sites (Fig. 1B). The same relationship was found with the number of adult *H. parallelus* on infested roots, with significantly more beetles on roots of trees with either recent or old attacks by *D. valens* than on infested roots on unattacked trees (Fig. 1A). The number of beetles on infested roots was lower on trees with recent attacks by *D. valens* than on trees with old attacks. There were no significant differences in total numbers of roots/tree among uninfested, newly-attacked and old-attacked trees at the Tunlanchuan and Yaopin Forest Stations (ANOVA, *F*_2, 27_ = 2.134, *P* = 0.34 and *F*_2, 27_ = 1.765, *P* = 0.41, respectively). The total numbers (±SD, *n* = 10) of roots/tree at the Tunlanchuan Forest Station were 5.12 (±3.01), 6.17 (±2.31) and 5.71 (±2.91) for unattacked, new-attacked and old-attacked trees, respectively. At the Yaopin Forest Station, the total numbers (±SD, *n* = 10) of roots/tree were 4.98 (±2.51), 6.57 (±3.04) and 5.89 (±2.77), respectively. We found some evidence of successful brood production by *H. parallelus* in roots of old-attacked trees, from attacks that occurred in the previous year. However, brood establishment was not quantified. Recurring attacks by *D. valens* were also evident in old-attacked trees.

### Volatiles Produced by *H. parallelus*

**Figure 2:**
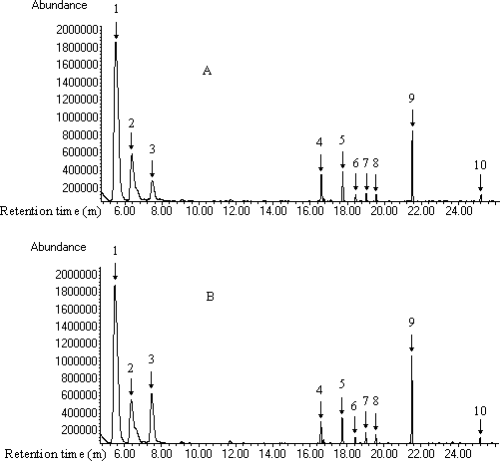
Typical gas chromatograms of male (A) and female (B) *H. parallelus* hindgut extracts. Each extract contains contents from 20 individuals. 1 α-pinene, 2 β-pinene, 3 limonene, 4 norinone, 5 myrtenal, 6 *trans*-verbenol, 7 cryptone, 8 *cis*-verbenol, 9 myrtenol, 10 isoborneol.

In experiment 2, the most prominent volatile in the hindguts of male and female *H. parallelus* was α-pinene, with mean (±SE) percentages of 58.4 (±1.0) and 58.4 (±1.2), respectively (*n* = 4) (Fig. 2). The next most common terpenes were β-pinene and limonene, with mean (±SE, *n* = 4) percentages of 15.5 (±1.0) and 10.7 (±1.3), respectively, in extracts from male hindguts, and 13.8 (±1.0) and 13.8 (±0.8), respectively, in extracts from female hindguts. Myrtenol, myrtenal and norinone accounted for 6.4 (±0.2), 3.4 (±0.2) and 2.6 (±0.3) percent, respectively, of volatiles in male extracts and 6.5 (±0.3), 3.2 (±0.3) and 1.9 (±0.1) percent, respectively, of volatiles in female extracts. In male and female extracts, *trans*-verbenol, cryptone, *cis*-verbenol and isoborneol were each present at ≤1%. 3-Carene was not detected in hindgut extracts. We found no evidence of any sex-specific production of compounds.

### Antennal and Walking Responses of *H. parallelus*

**Figure 3 pone-0001302-g003:**
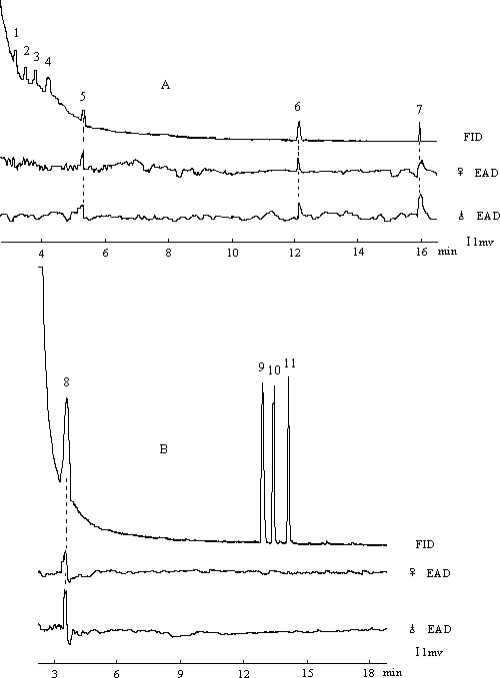
Typical EAD responses of *H. parallelus* antennae to blend A (A) and B (B). 1 (+)-α-pinene, 2 (−)-β-pinene, 3 myrcene, 4 (−)-limonene, 5 terpinolene, 6 (±)-myrtenal, 7 (±)-myrtenol, 8 (+)-3-carene, 9 (+)-*trans*-verbenol, 10 (+)-*cis*-verbenol, 11 verbenone.

The electroantennographic detector (EAD) responses of *H. parallelus* in experiment 3 were comparable among all ten male and ten female antennae that we tested. Typically terpinolene, (±)-myrtenal and (±)-myrtenol elicited responses by male and female antennae of *H. parallelus* (Fig. 3A, compounds 5, 6 and 7, respectively), with little, if any, response to (+)-α-pinene, (−)-β-pinene, myrcene or (−)-limonene. Similarly, (+)-3-carene elicited responses in both male and female antennae (Fig. 3B, compound 8), with little, if any, EAD response to (+)-*trans*-verbenol, (+)-*cis*-verbenol and verbenone. There were no significant differences between sexes in antennal responses to (+)-3-carene, terpinolene, (±)-myrtenal and (±)-myrtenol (Mann-Whitney test, *P* = 0.728, *P* = 0.376, *P* = 0.459, and *P* = 0.241, *n* = 10, respectively).

**Figure 4 pone-0001302-g004:**
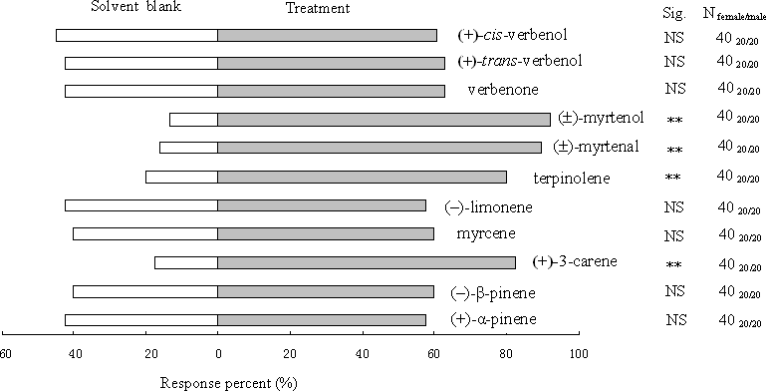
Walking responses of *H. parallelus* compounds in Y-tube olfactometer trials. **, significant differences at *P*<0.01. *n* = 40 (20 male: 20 female) responding beetles for each treatment.

The walking responses of *H. parallelus* in experiment 4 were similar to the EAD responses in experiment 3. Male and female *H. parallelus* were attracted to (+)-3-carene, terpinolene, (±)-myrtenal, and (±)-myrtenol (Fig. 4). Walking responses were unaffected by the common monoterpenes, (+)-α-pinene, (−)-β-pinene, myrcene and (−)-limonene, and the terpene-derived compounds, (+)-*trans*-verbenol, (+)-*cis*-verbenol and verbenone.

### Interspecific Responses of *D. valens*

**Figure 5:**
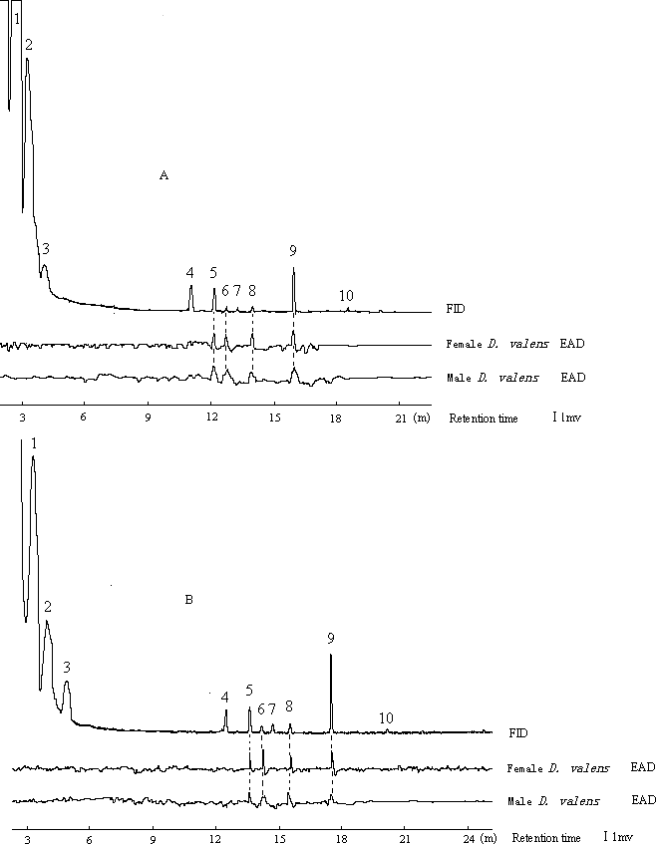
Typical EAD responses of *D. valens* antennae to male and female *H. parallelus* hindgut extracts. 1 α-pinene, 2 β-pinene, 3 limonene, 4 norinone, 5 myrtenal, 6 *trans*-verbenol, 7 cryptone, 8 *cis*-verbenol, 9 myrtenol, 10 isoborneol.

**Figure 6:**
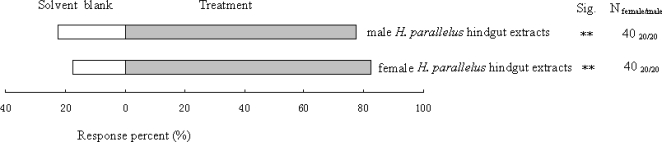
Walking responses of *D. valens* to *H. parallelus* hindgut extracts in Y-tube olfactometer trials. **, significant differences at *P*<0.01. *n* = 40 (20 male: 20 female) responding beetles for each treatment.

The EAD responses of antennae from *D. valens* in experiment 5 were consistent among all antennae that we tested. Typically, *trans*-verbenol, myrtenol, *cis*-verbenol and myrtenal in hindgut extracts from *H. parallelus* elicited EAD responses from *D. valens* (Fig. 5), with no significant differences between the sexes (Mann-Whitney test, *P* = 0.479, *P* = 0.598, *P* = 0.794, and *P* = 0.198, *n* = 10, respectively). In experiment 6, male and female *D. valens* were significantly attracted to male and female *H. parallelus* hindgut extracts in the Y-tube olfactometer (Fig. 6).

## Discussion

Various factors have likely led to facilitation among bark beetles. Natural selection should favor bark beetles that can locate and occupy breeding niches in a timely manner since the nutrient quality of phloem tissue can desiccate and degrade quickly [26]. The selective advantage for utilizing these nutrient-rich opportunities seem clear as breeding opportunities for bark beetles tend to be patchy and unpredictable [27]. Chemical ecology is an important part of the facilitation between bark beetle species, allowing individuals to quickly locate breeding opportunities yet maintain separation of species [11]. In the southern United States, several species of bark beetles are often found in association with the tree-killing species, *Dendroctonus frontalis* Zimm., with clear separation of niches on loblolly pine, *Pinus taeda* L. [28]. Two secondary species of bark beetles, *Ips avulsus* Eichh. and *I. calligraphus* Germ. arrive on loblolly pines attacked by *D. frontalis* within 1–3 days of attacks by *D. frontalis*, facilitated by considerable chemical cross-attraction among these species [29]–[31].

In spite of no evolutionary history, the same type of association (mediated by semiochemicals) seems to be occurring in China with the exotic *D. valens* and the native *H. parallelus* in *P. tabuliformis. Dendroctonus valens* seems to prefer the lower trunk and upper roots of *P. tabuliformis* whereas *H. parallelus* invades the remaining root system, allowing both species to attack the same trees simultaneously. In trees attacked by *D. valens*, 20–40% of the roots were also infested by *H. parallelus* (Fig. 1) with both species re-attacking the same trees over a period of 2–3 years.

The association between the exotic *D. valens* and the native *H. parallelus* in *P. tabuliformis* is likely not random, as very few trees were attacked solely by *H. parallelus* (Fig. 1). The host volatile, (+)-3-carene is abundant in hindgut volatiles of *D. valens* [25]. The antennae of both species are sensitive to (+)-3-carene (Figs. 3 and 5) [25]. In walking bioassays, both species are attracted by (+)-3-carene. (Fig. 4). In field studies, traps baited with (+)-3-carene are attractive to flying *D. valens* [14]. Responses of flying *H. parallelus* to (+)-3-carene have yet to be determined. In addition, both species exhibit electroantennographic detector (EAD) and walking responses to two compounds produced by both species, (±)-myrtenol and (±)-myrtenal (Figs. 2–5) [25]. Moreover, walking *D. valens* are attracted by volatiles produced by *H. parallelus* (Fig. 6).

In this study, the newly formed relationship between the exotic *D. valens* and the native *H. parallelus* has facilitated aggregation of *D. valens* on their shared host in China. Aggregation is the primary process for establishment and dispersal of bark beetles [32]. Therefore, this facilitative interactions between an exotic (*D. valens*) and a native (*H. parallelus*) bark beetle may help explain how the exotic bark beetle (*D. valens*) invades the non-native area (China).

Factors other than facilitation may be involved in the invasiveness of *D. valens* in China. The hypotheses of empty niche and enemy release for invasive species, respectively [1], [33], may be applicable as competing species of bark beetles and associated predators and parasitoids are lacking in China [13]. Evolution of improved competitive ability [6] by *D. valens* may also play a role in China. In China, most *D. valens* breed below the root collar, thereby ensuring high survivorship over the winter months [34]. Efforts should also be made to determine the association of fungi with both *H. parallelus* and *D. valens. Leptographium terebrantis* Barras & Perry, a fungal associate of *D. valens* and other bark beetles in North America, is associated with decline and mortality of various species of pines and bark beetles [35]–[38].

Further work is required to determine the role of facilitation and other invasion mechanisms for *D. valens* in China. The reproductive success of both species, together and separate in *P. tabuliformis* needs to be determined. The availability of natural breeding opportunities for *H. parallelus* in the absence of *D. valens* needs to be determined as well. It is possible that the activities of *D. valens* in China provide *H. parallelus* with greater opportunities than normal for breeding purposes.

Our results also suggest that the risk from exotic species may not be limited to those species that are aggressive in their native ranges. With the use of semiochemicals, two relatively benign species (the exotic *D. valens* and the native *H. parallelus*) may act in concert to overcome tree defenses. Recently, an exotic ambrosia beetle from Asia, *Xyleborus glabratus* Eichhoff, was introduced into the coastal region of southeastern United States. In a fashion similar to Dutch elm disease, the association of *X. glabratus* with a wilt disease has caused extensive mortality of redbay, *Persea borbonia* (L.) Spreng, from Jacksonville, Florida, to Charleston, South Carolina, within a span of only five years [39]. Neither species had previously been known to cause damage in any other country. So risk assessments for invasive species need to consider diverse potential scenarios and not simply focus on species that are detrimental in their native ranges. Similarly, management programs for invasive species will have to be highly innovative and adaptable in reducing such threats.

Some reported invasive mechanisms, such as invasional meltdown, focus on the interspecific facilitation between introduced species [8], [40]. However, our results report, for the first time, facilitative relationship between an exotic and a native bark beetle seems to involve overlap in the use of host attractants and pheromones. Mondor and Addicott also showed that cross-species communication exists between the invasive Argentina ant, *Linepithema humile* Mayr and native poplar aphid, *Chaitophorus populicola* Thomas [24]. In addition, such interactions, which may well be possible for many other secondary pest groups, may be of great benefit in predicting invasiveness of these heretofore low-visibility pests. As the further step, more examples on facilitation between exotic and native insects should been investigated, which could be explored as a novel invasive mechanism for not only exotic bark beetles but also other introduced pests.

## Materials and Methods

### Field Association between *D. valens* and *H. parallelus*

In experiment 1, we assessed the association between *D. valens* and *H. parallelus* in plantations of *P. tabuliformis* at two locations in China: (1) the Tunlanchuan Forest Station (N 37° 48′, E 111° 44′; average, elevation 1400 m), west of the city of Gujiao, Shanxi Province; and (2) the Yaopin Forest Station (N 35° 46′, E 109° 16′; average, elevation 1000 m), Shaanxi Province. The plantations were 35 and 40 years in age, respectively, with mean tree diameters at breast height of 23.2 cm and 24.4 cm, respectively.

At each location, we randomly selected ten pine trees (at least 20 m apart) with no attacks by *D. valens*, ten pine trees with new attacks by *D. valens* in 2006 and 10 pine trees with old attacks by *D. valens* from 2005. Trees attacked in 2005 had fading foliage, yellow in color, whereas unattacked and recently-attacked trees had green foliage. In October 2006, each tree was excavated from the stem base along the roots for a length of about 1.5 m. For each tree, we recorded the total number of main roots and the number of roots under current attack by *H. parallelus* as well as the total number of adult *H. parallelus* on each infested root. The term “infested” is used simply to differentiate attack activities of *H. parallelus* from those of *D. valens* and is not meant to imply successful establishment of brood galleries. Successful brood establishment was not assessed for either species in this study.

### Volatiles Produced by *H. parallelus*

In all laboratory experiments, we used *D. valens* and *H. parallelus* that emerged from naturally-infested *P. tabuliformis* collected at the Tunlanchuan Forest Station. Beetles were sexed and maintained in an incubator at 25°C and 55% RH, under a light regime of 14L:10D prior to use in experiments. Semiochemical compounds used for all experiments were purchased from Pherotech International Inc. (Delta, British Columbia, Canada) and included the following compounds which were known to be associated with pine hosts or *D. valens* [25]: (+)-α-pinene, (−)-β-pinene, myrcene, (−)-limonene, (+)-3-carene, terpinolene, (±)-myrtenol, (+)-*trans*-verbenol, (+)-*cis*-verbenol, (±)-myrtenal and verbenone (chemical purities, 85, 86, 80, 84, 87, 86, 95, 84, 87, 95 and 98%, respectively). The enantiomeric composition of verbenone was 66% (+) and 34% (−).

In experiment 2, we determined the composition of volatiles in the hindguts of *H. parallelus.* The hindguts of 20 male and 20 female *H. parallelus* were transferred to separate glass vials containing 4 ml hexane. The extracts were filtered through glass wool and stored in a freezer (−10°C) until analyzed. This extraction procedure was repeated three times, resulting in four extracts representing 80 beetles for both male and female *H. parallelus* (*n* = 4). Prior to analysis, hindgut extracts for each sex were diluted to 200 µl. Aliquots of extracts (2.5 µl) were injected splitless into a gas chromatograph–mass spectrometer (GC–MS) (Hewlett Packard 6890N GC model coupled with 5973 MSD), equipped with a DB-WAX column (60 m length×0.25 mm i.d.×0.25 µm film) (J&W Scientific, Folsom, CA, USA). The GC oven temperature program was set at 50°C for 2 min; increased to 220°C at 5°C/min; increased to 230°C at 4°C/min; and set at 230°C for 5 min. The on-column injector temperature was 220°C and helium was the carrier gas (flow rate, 1ml/min). The mass spectrometer (MS) electron impact source was operated in scan mode (30–300 amu) with the MS source temperature at 230°C and the MS Quad at 150°C. Identifications of chromatogram peaks were based on comparisons with retention times and mass spectra of known standards and those in the NIST02 library (Scientific Instrument Services, Inc., Ringoes, NJ, USA). Enantiomeric compositions of volatiles were not determined.

### Antennal Responses of *H. parallelus*

In experiment 3, we examined the coupled gas chromatography-electroantennographic detector (GC-EAD) [41] responses of *H. parallelus* antennae to various semiochemicals associated with *H. parallelus* (experiment 2) and *D. valens* [25]. The procedure allows separation of individual compounds in blends prior to simultaneous exposure of individual compounds to a flame ionization detector (FID) and an electroantennographic detector (EAD). Each EAD was made by cutting off the tip of an excised antenna and then mounting the antenna between two glass micropipette electrodes, filled with Kaissling saline. The recording electrode was positioned at the distal edge of the antennal club whereas the reference electrode was positioned near the scape. The electrodes were held with micromanipulators (Syntech MP15; Syntech, Hilversum, the Netherlands) and connected to a high impedance input AC/DC amplifier model UN-06 (Syntech) through Ag/AgCl junctions. Amplified GC-EAD responses were digitized using a Nelson 900 Series Interface, and displayed and processed using AutoSpike software (Syntech).

In order to minimize overlap in GC retention times, two blends of compounds were used in GC-EAD determinations. Blend A consisted of (+)-α-pinene, (−)-β-pinene, myrcene, (−)-limonene, terpinolene, (±)-myrtenol, and (±)-myrtenal (1 mg of each), and diluted to 5 ml hexane. Blend B consisted of (+)-3-carene, (+)-*cis*-verbenol, (+)-*trans*-verbenol and verbenone (10 mg of each), and diluted to 10 ml hexane. For each EAD, an aliquot (1 µl) of one of the two blends was injected, splitless into a HP 6890 gas chromatograph, equipped with a DB-WAX column (30 m×0.25 mm×0.25 µm film thickness) (J&W Scientific, Folsom, CA, USA) using the following temperature program: initially set at 80°C; increased to 200°C at 5°C/min; increased to 230°C at 6°C/min. The carrier gas was helium (flow rate, 1.5 ml/min). The injector and detector temperatures were 220°C and 240°C, respectively. At the end of the column, a GC effluent splitter (press-fit connection; split ratio 1∶1) was used to supply the FID and an EAD. For the EAD, effluent from the GC column was added to a purified and humidified air stream, passed over the excised antenna. Each blend was tested separately on antennae from ten male and ten female *H. parallelus*.

### Walking Responses of *H. parallelus*

In experiment 4, we assessed the walking responses of *H. parallelus* to the same compounds tested on antennae in experiment 3, with each tested separately. Walking responses were assessed in a glass Y-tube olfactometer (35-mm diameter by 40 cm long, with a 120° inside angle) with airflow at 200 ml/min through each branch. Incoming air was filtered through activated charcoal and humidified with double distilled, de-ionized water. The filtered air was split between two holding chambers: one chamber served as a control (solvent blank) and the other chamber held the test material. Test chemicals (100 µg in 10 µl hexane) were applied to a paper filter strip (5 by 50 mm) that was placed in one of the two holding chambers after the solvent had been allowed to evaporate for 20 sec. Air passed from each holding chamber into the respective branches of the Y-tube. A smoke test verified laminar airflow in both branches and throughout the olfactometer.

Approximately 30 min before trials were initiated, adult *H. parallelus* were placed into individual holding/release tubes and isolated from possible semiochemical sources. At the beginning of each trial, a single beetle was placed at the down-wind end of the Y-tube. Each beetle was given 10 min to respond, with the choice of left or right branches of the olfactometer noted when the beetle walked 5 cm past the Y-tube junction. The olfactometer was maintained at 25°C and 70% RH during trials. Treatments associated with the right and left branches of the olfactometer were exchanged after every fifth beetle. Y-tubes replaced with clean ones when treatments were changed or positions exchanged. Individual bark beetles were tested only once.

### Interspecific Responses of *D. valens*

In experiment 5, we determined the interspecific EAD responses of *D. valens* to hindgut extracts from *H. parallelus*. One aliquot of extract (2.5 µl), derived from one male *H. parallelus* hindgut, was injected into the GC using the same protocol noted above in experiment 3, with *D. valens* antennae used for the EAD. Each extract was presented to ten male and ten female *D. valens*. The procedure was repeated with hindgut extracts from individual female *H. parallelus*.

In experiment 6, we assessed the walking responses of *D. valens* to volatiles produced by *H. parallelus* using the same protocol noted in experiment 4. The test chemicals were extracts of male and female *H. parallelus* hindguts. In each trial, one aliquot (2.5 µl) of either male or female hindgut extracts (equivalent to the hindgut contents of a single beetle) was applied to a paper filter strip and deposited into one of the two holding chambers of the Y-tube olfactometer.

### Statistical analyses

All data were analyzed with SPSS 11 for Windows [42]. One-way ANOVA was used to compare the differences among roots of different attack categories and average number of *H. parallelus* on each attacked root in experiment 1. Differences between the sexes in amplitude of EAD responses in experiments 3 and 5 were analyzed using Mann-Whitney tests. In experiments 3 and 6, the null hypothesis that beetles showed no preference for either olfactometer arm (and thus showed no response to test compound) was tested using a table of cumulative binomial probabilities with a *p*-value of 0.05. We used Chi ^2^ tests to compare differences between sexes in walking responses in olfactometer.
